# Arteriovenous fistulas maturation: predictors of maturation and use of ultrasound

**DOI:** 10.1590/2175-8239-JBN-2023-E011en

**Published:** 2023-09-25

**Authors:** Wagner Moura Barbosa, Ricardo Portiolli Franco, Anderson Tavares Rodrigues

**Affiliations:** 1 Universidade de Pernambuco, Faculdade de Ciências Médicas, Recife, PE, Brazil.; 2 Fundação Pró-Renal Brasil, Curitiba, PR, Brazil.; 3 Pró-Renal Centro de Nefrologia, Barbacena, MG, Brazil.

The creation and maturation of arteriovenous fistulas (AVF) for hemodialysis (HD) are crucial to maintain an effective dialysis therapy. The failure of AVF maturation is a daily obstacle for specialists dedicated to vascular accesses for HD; however, despite technological and technical developments, the correct prediction of maturation is still limited.

In this context, preoperative mapping with ultrasound (US) has been a routine ally in identifying vessels that provide a greater chance of maturation, and in planning future vascular accesses. In clinical practice, this evaluation takes place in several ways, from point of care approaches in the HD clinic, formal evaluations by radiologists who send reports to surgeons, even in the immediate preoperative period, performed by the surgeon himself. In any of these scenarios, the US can add clinically relevant information and change treatment procedures.

Recognition of the high incidence of maturation failure and better understanding of it led to modifications in the latest KDOQI vascular access guidelines. The 2019 update suggests seeking the right access for the right patient, for the right reason, as well as planning life and accesses during dialysis treatment. This means that, although arteriovenous grafts have their place as the first choice for a certain group of patients, such as those with low life expectancy on HD or with inadequate vascular territories, a native AVF that reaches clinical maturation is still the best access for most patients. The 2019 KDOQI guidelines suggest the use of preoperative US in patients with risk factors for maturation failure and comment on the lack of strong evidence in favor of the universal use of US^1^.

In this issue of the BJN, Gasparin et al.^2^, in a prospective observational trial, sought to assess the association of clinical and sonographic factors with the maturation of AVF created in an outpatient surgical center. Maturation in 4 to 6 weeks, defined by venous diameter greater than 0.40 cm and flow volume greater than 500 mL/min on Doppler, was 77.9%. Factors associated with maturation in the multivariate analyzes were skinfold thickness (OR 0.32), arm circumference (OR 0.83), current or past smoking (0.35), and vein diameter greater than 0.36 cm (OR 4.89). In the sample, 36.5% of the AVF were radiocephalic and 46.2% were brachiocephalic. The authors argue that the above-average maturation rate may be due to the US used in the preoperative period, enabling the selection of the best vessels for anastomosis.

In 2002, a retrospective study found a 16%, maturation rate for radiocephalic AVF, when the smallest diameters of the cephalic vein were below 0.2 cm; and of 76%, when above this measurement^3^. This study also demonstrated the importance of assessing the vessel as a whole, up to its discharge into the central circulation, and that the assessment of diameters only in a possible anastomosis may be insufficient. In the case of radiocephalic AVFs, other factors must be considered, such as venous distensibility and reactive hyperemia of the radial artery on the Doppler^4,5,6^, since Wilmink and Corte-Real Houlihan^7^ demonstrated that the diameter of the vessels alone is a poor predictor of the functional use of AVFs. Another important consideration regarding US is its use for postoperative flow assessment as a predictor of failure. In 2018, Benaragama et al.^8^ reported that flows below 300 mL/min in the postoperative period identify AVFs with a high risk of early failure and, therefore, these patients may be candidates for early intervention with alternative approaches, for example, balloon-assisted percutaneous maturation. In addition to postoperative flow, the AVF diameter and depth are variables that moderately predict unassisted clinical maturation, as well as overall AVF survival^9^.

More recently, a machine learning model was proposed, using mainly US data, capable of predicting the maturation of AVFs with 96.8% accuracy. The authors suggest that this algorithm could be implemented directly in the US devices, without requiring additional time for the calculation^10^. This could even correct the interobserver bias that Gasparin et al.^2^ consider existing in ultrasound venous mapping tests.

It is not clear whether the mapping performed by an external examiner would have a different impact than the examination performed by the surgeon in the immediate preoperative period. In the most basic US point of care assessments, it is possible to assess venous diameters and decide whether or not to refer a particular patient for making an arteriovenous access. In more comprehensive assessments, including arterial Doppler parameters, additional data may change the approach regarding the type of AVF to be created.

The possibility of identifying more favorable vessels for AVF maturation and the excellent Gasparin et al. maturation rate reinforces the importance of US, although we still need more prospective studies evaluating the use of US in the preoperative period.
